# Testing the effectiveness of a transdiagnostic treatment approach in reducing violence and alcohol abuse among families in Zambia: study protocol of the Violence and Alcohol Treatment (VATU) trial

**DOI:** 10.1017/gmh.2017.10

**Published:** 2017-10-02

**Authors:** J. C. Kane, S. Skavenski Van Wyk, S. M. Murray, P. Bolton, F. Melendez, C. K. Danielson, P. Chimponda, S. Munthali, L. K. Murray

**Affiliations:** 1 Department of Mental Health, Johns Hopkins Bloomberg School of Public Health, 624 North Broadway, Room 850, Baltimore, MD 21205, USA; 2 Department of International Health, Johns Hopkins Bloomberg School of Public Health, 615 North Wolfe Street, Baltimore, MD 21205, USA; 3 Department of Psychiatry and Behavioral Sciences, Medical University of South Carolina, 67 President St, Charleston, SC 29425, USA; 4 Serenity Harm Reduction Programme Zambia (SHARPZ), Plot # 220C, Mutandwa Road, Roma, Lusaka, PO Box 33705, Zambia

**Keywords:** Alcohol, common elements treatment approach, interventions, intimate partner violence, low- and middle-income country, randomized clinical trial

## Abstract

**Background:**

Violence against women and girls (VAWG) is an urgent global health problem. Root causes for VAWG include the individual- and family-level factors of alcohol abuse, mental health problems, violence exposure, and related adverse experiences. Few studies in low- and middle-income countries (LMIC) have assessed the effectiveness of psychological interventions for reducing VAWG. This randomized controlled trial, part of the *What Works to Prevent Violence Against Women and Girls* consortium, examines the effectiveness of a common elements treatment approach (CETA) for reducing VAWG and comorbid alcohol abuse among families in Zambia.

**Methods/design:**

Study participants are families consisting of three persons: an adult woman, her male husband or partner, and one of her children aged 8–17 (if available). Eligibility criteria include experience of moderate-to-severe intimate partner violence by the woman and hazardous alcohol use by her male partner. Family units are randomized to receive CETA or treatment as usual. The primary outcome is VAWG as measured by the Severity of Violence Against Women Scale, assessed along with secondary outcomes at 24 months post-baseline. Interim assessments are also conducted at 4–5 months (following CETA completion) and 12 months post-baseline.

**Conclusions:**

This ongoing trial is one of the first in sub-Saharan Africa to evaluate the use of an evidence-based common elements approach for reducing VAWG by targeting a range of individual- and family-level factors, including alcohol abuse. Results of this trial will inform policy on what interventions work to prevent VAWG in LMIC with local perspectives on scale up and wider implementation.

## Background

Violence against women and girls (VAWG) is a recognized global health and human rights problem. In 2010, approximately one in three adult women were estimated to have experienced either physical or sexual intimate partner violence (IPV) in their lifetime (Devries *et al.* 2013). For a substantial proportion of these women, the physical violence experienced is severe, and poly-victimization is common (Garcia-Moreno *et al.* 2006). A recent systematic review of population-based surveys identified that approximately half of children in Asia, Africa, and North America experienced some form of violence in the past year, with violence in the home the predominant form for children between the ages of 2 and 14 (Hillis *et al.* 2016). Global prevalence estimates indicate that child sexual abuse victimization occurs in approximately one out of every eight children (Stoltenborgh *et al*. 2011). In a multi-national study, Zambia, the location of the present study, was found in a multi-national study to have the highest percentage of ever married women reporting IPV (48%) (Kishor & Johnson, 2004), and our own studies indicate that a substantial percentage of youth are exposed to a high number and wide range of violence experiences (Murray *et al.* 2006, 2015).

Violence has many repercussions. IPV in women is associated with severe injury, gynecological problems, chronic pain, increased risk of sexually transmitted infections (including HIV), poorer overall self-rated health, alcohol use, post-traumatic stress, depression, anxiety, and suicidal ideation, with more severe symptoms linked to poly-victimization (Campbell, 2002; Ellsberg *et al.* 2008; Trevillion *et al.* 2012; Dillon *et al.* 2013; Devries *et al.* 2014; Lagdon *et al.* 2014; Li *et al.* 2014). Experiencing abuse and witnessing violence as a child are associated with a wide range of negative outcomes, including injury, developmental deficits, risk-taking behaviors, poor physical health, mental health problems, and death (Campbell & Lewandowski, 1997; Felitti *et al.* 1998; Springer *et al.* 2007; Irish *et al.* 2010). Further, research shows that witnessing or experiencing violence within the home predicts victimization and perpetration of violence in later life (Abramsky *et al.* 2011; Fulu *et al.* 2013; Fonseka *et al.* 2015).

Partner alcohol abuse is a strong predictor of experiencing IPV (Abrahams *et al.* 2004; Jewkes *et al.* 2006; Capaldi *et al.* 2012). Women whose husbands frequently return home drunk have an increased risk of experiencing abuse (Abramsky *et al.* 2011; Fulu *et al.* 2013). The role of alcohol use in IPV perpetration and experience is a particular concern in low- and middle-income countries (LMIC), specifically in sub-Saharan Africa, where rates of problematic alcohol use are increasing (Shield *et al.* 2013). Mental health problems, particularly depression and post-traumatic stress disorder, have also been associated with experiencing (Iverson *et al.* 2011; Kuijpers *et al.* 2012) and perpetrating (Fulu *et al.* 2013; Oram *et al.* 2014) violence.

Economic and social ‘structural’ interventions have demonstrated effectiveness for addressing some risk factors (e.g. inequitable gender norms) of violence, but findings for actual reductions in its occurrence are mixed (Bourey *et al.* 2015). Although these structural interventions are essential to broad primary prevention of IPV, the complex inter-relationship between violence, alcohol, and mental health suggests a need for integrated approaches that include treatments targeting high-risk groups (Guedes *et al.* 2016). Two trials conducted in the USA suggest cognitive–behavioral therapy (CBT) interventions and alcohol treatment combined with violence prevention programs show promise for impacting some types of IPV (Tirado-Muñoz *et al.* 2014; Wilson *et al.* 2014). Given the high rate of IPV in LMIC and the dearth of evidence-based interventions in these settings, more studies are needed to evaluate approaches that address high-risk families affected by IPV, alcohol use, and/or mental health problems.

This paper describes: (1) the process of adapting an evidence-based, modular transdiagnostic treatment approach (common elements treatment approach; CETA) (Murray *et al.* 2014) to address violence and alcohol abuse, and (2) the protocol for testing CETA in an ongoing randomized controlled trial. The trial is part of a larger consortium of studies, the *What Works to Prevent Violence Against Women and Girls Programme* (http://www.whatworks.co.za/), investigating the underlying causes of VAWG and strategies for prevention in LMIC. The primary aims of the trial are to evaluate the effectiveness of the adapted CETA intervention on (a) reducing and preventing women's experience of IPV and (b) reducing male partner's hazardous alcohol use.

## Methods/design

### Overview of study design

**Fig. 1.**
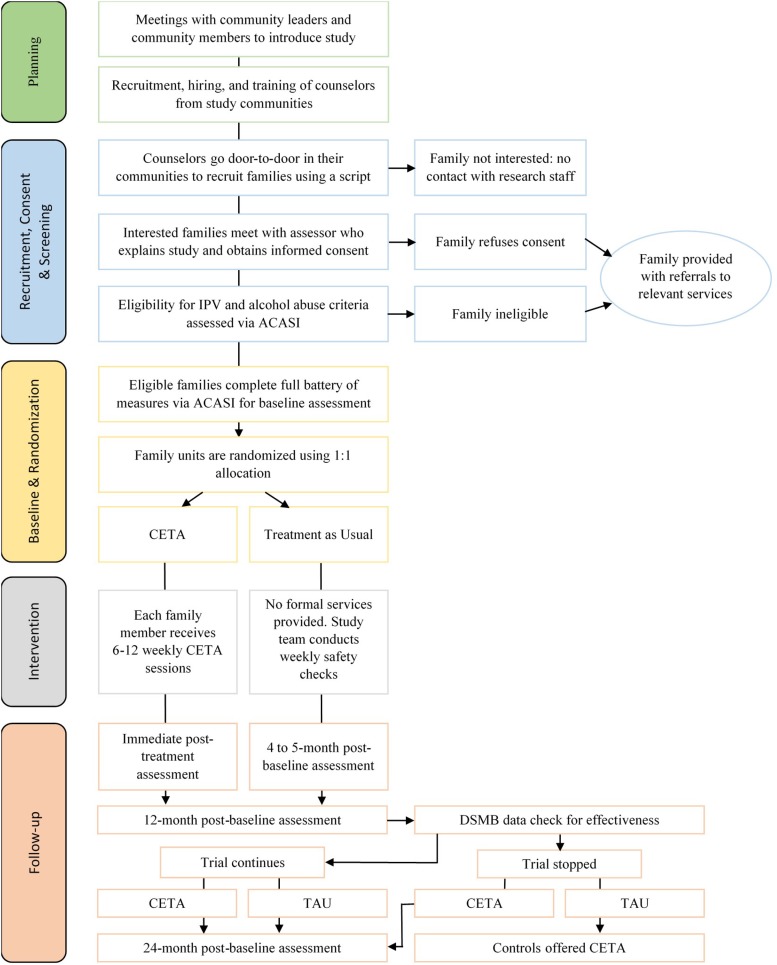
Flow of study procedures for VATU trial.

The Violence and Alcohol Treatment (VATU) study (*vatu* means ‘ours’ in Nyanja, a commonly spoken language in Zambia) is a parallel group randomized clinical trial comparing the effectiveness of CETA to treatment as usual (TAU) among family ‘units’ consisting of three individuals: an adult woman, her male husband or partner, and one of her children (male or female, ages 8–17). The study settings are three high-density, low-resource ‘compounds’ (i.e. neighborhoods) in Lusaka. Eligible families are randomized as a unit to CETA or to TAU. All participants are assessed at four time points: (1) baseline; (2) 4–5 months (following CETA completion); (3) 12 months; and (4) 24 months post-baseline. The study flow is illustrated in Fig. 1.

### Ethical approval and trials registration

The study has been approved by the Johns Hopkins Bloomberg School of Public Health Institutional Review Board and the University of Zambia Biomedical Research Ethics Committee. The trial is registered at ClinicalTrials.gov (NCT02790827; date of registration 05/24/2016). The study methods described below cover the recommended items to address in a clinical trial protocol according to SPIRIT (Chan *et al.* 2013).

### Recruitment

Initial recruitment, screening, baseline assessment, and randomization for the study were conducted between May and July 2016. Following principles of community-based participatory research, our research staff met with community leaders in each compound to discuss the purpose and procedures of the trial prior to its initiation and organized larger meetings open to the community where questions and concerns about the study were addressed. We have found in previous studies in Zambia that this type of community involvement is essential to success (Murray *et al.* 2006; Kane *et al.* 2016*b*). This approach also enabled collaboration with local leaders in identifying community members who they felt were suitable candidates for being hired and trained as CETA counselors. Recruitment of study participants was subsequently conducted by these study counselors. This provider-based recruitment strategy was employed because it is the current method for outreach used by our partnering organization in Zambia for similar behavioral interventions and it has been found to be culturally acceptable and feasible for potentially sensitive topics, such as alcohol use.

Counselors received a 1-day training in recruitment procedures before going door-to-door in their communities with a script to assist in explaining the purpose of the study to families. Counselors worked in male/female pairs when visiting families’ homes, so that women could speak alone to a counselor of the same sex. To ensure that participation in the study would not exacerbate or lead to IPV, counselors specifically asked the women about possible risks. If the woman expressed concern for her safety, the counselor offered her information on, or assistance in, accessing services. Women with a child between the ages of 8–17 were asked if they would like to provide permission for the child to participate. Women with more than one child were encouraged to select the child whom they perceived as being most affected by the violence in the home. Families without a child in this age range or those that did not want a child to participate were still able to join the study as a couple.

### Eligibility screening

Families who expressed interest in the study were invited to participate in an eligibility screening at a later date, typically within 1 week, at a site in their community (i.e. school, church, or community center). Study assessors met with families privately and provided a brief overview of the purpose and procedures of the study to all family members (i.e. the woman, man, and child). Informed consent was then obtained with each family member separately in a private space. Consent was obtained for all study activities (screening, assessments, and intervention).

Following informed consent, the woman and her male partner separately completed an eligibility screener. For women, this consisted of the Severity of Violence Against Women Scale (SVAWS; Marshall, 1992), for assessing recent IPV; and the Alcohol Use Disorders Identification Test (AUDIT; Saunders *et al.* 1993), for measuring her report of her partner's hazardous drinking. Men only completed a self-reported AUDIT. To be eligible, (a) the woman had to report recent (past 12 months) experience of at least a moderate level of violence (SVAWS physical violence subscale score of ⩾38) and (b) she or her partner had to report that he has engaged in hazardous alcohol use (AUDIT score of ⩾8). Additional eligibility criteria are detailed in Box 1.

Box 1. Inclusion and exclusion criteria for the VATU trial.

Inclusion criteria

1. The family must live in one of the three study compounds in Lusaka (i.e. cannot only be staying temporarily).
2. All family members must speak at least one of the three study languages: English, Bemba, or Nyanja.
3. The family must consist of an adult (aged 18 years or older) female and adult male in a relationship (i.e. married or dating). In addition, the woman has the choice (if applicable) to identify one of her children between the ages of 8 and 17 for inclusion in the study.
4. Both the adult female and her male partner must provide consent. For one of her children to also participate, the woman's permission and the child's assent are required.
5. The adult female must score 38 or more on the Severity of Violence Against Women Scale (SVAWS; physical violence subscale) indicating experience of at least a moderate amount of intimate partner violence.
6. The adult male must have a score of 8 or higher on the Alcohol Use Disorders Identification Test (AUDIT) as reported by himself or his partner, indicating that he drinks alcohol at hazardous levels.

Exclusion criteria

1. No family member can currently be on an unstable (i.e. altered in last 2 months) psychiatric drug regimen.
2. No family member has had a suicide attempt or suicidal ideation accompanied by intent, a plan, or self-harm in the past month.
3. No family member has been diagnosed with a current psychotic disorder (identified by the University of Zambia Teaching Hospital Psychiatric Unit).
4. No family member has a serious developmental disorder (e.g. mental retardation, autism) that would preclude participation in a cognitive-behavioral oriented skills intervention or completing the assessment battery.

The screener was administered using a laptop-based Audio Computer Assisted Self-Interviewing (ACASI) system. ACASI was chosen for use because previous studies have found that it elicits more valid responses to questions on sensitive behaviors than face-to-face interviewing and because it is feasible and acceptable among this study population (Langhaug *et al.* 2010; Kane *et al.* 2016*a*, *b*). ACASI scored the screener immediately, and if the family was determined to be eligible, the assessor administered the full assessment battery (approximately 2 additional hours total; see Assessments section below) separately to all participating family members. If the family was ineligible, the assessor discussed services in the community that may be appropriate. Following administration of the full ACASI, any participant who reported suicidal ideation, homicidal ideation, or who was currently experiencing violence, was flagged as ‘high risk’. ACASI automatically alerted the assessor about the high-risk status, and the assessor immediately activated a standardized safety plan (included in full as online Supplementary material and summarized in Box 2).

Box 2. Summary of VATU study participant safety protocol.

- Assessing and responding to suicide risk
  - Evaluate suicide risk
    - Are you thinking about killing yourself or ending your life?
    - Have you ever tried to kill yourself or end your life?
    - Do you have a plan to kill yourself or end your life?
    - Do you have access to that plan; in other words, do you have the means to execute your plan?
  - Response
    - If the client answers yes to any of these questions call your supervisor immediately while the client is still working with you. Decide and agree on a plan before the client leaves.
    - Safety planning may include creating a safety contract with the client, identifying warning signs and coping skills, using safety watch and imminent risk referrals to the closest facility where the client can be monitored more closely.
- Assessing and responding to homicide risk
  - Assessing homicide risk
    - Have you ever tried to end someone's life/hurt someone before?
    - Are you thinking about ending someone's life/hurting someone?
    - Do you have a plan to end someone's life/hurt that person?
    - Do you have access to that person; in other words, do you have the means to execute your plan?
  - Response
    - If a client answers ‘YES’ to any of these questions, immediately inform the clinical supervisors. Staff will arrange for the person to be accompanied to a safe place, and will call her/his supervisor ahead to advise the supervisor of the situation.
    - Safety planning may include safety contracts, safety watch, and where needed reporting to local authorities and referral for observation.
- Assessing and responding to interpersonal violence risk
  - Evaluate interpersonal violence risk
    - Is the person being violent in the home (e.g. hitting, kicking, etc.) living with you?
    - In what ways has this person hurt you?
    - In what ways do you think this person could hurt you?
  - Response
    - The staff will talk to her/his supervisor while the participant is still working with them. The staff and the participant will decide or agree on a plan BEFORE the participant leaves the session.
    - Safety planning varies based on local laws but may include reporting to victim support unit of the police, referral to a shelter or gender based violence one-stop center or safety prevention plans in the home.
- Assessing and responding to child abuse risk
  - Evaluate child abuse risk
    - Do you know the person who is abusing you?
    - Does the abuser stay in the same house with you?
    - Have you reported to anybody?
    - If you have reported, what has been done?
  - Response
    - If the client answers ‘YES’ to the first three questions, immediately call designated contacts at Social Welfare and the Child Protection Unit of the police. The staff shall ensure to call their supervisor at the end of the session before the client leaves.
    - If the client says no to the first questions ask for more information. For example: When was the last time the abuse happened? Where is the person who was abusing you currently staying? Does anyone in the home know about the abuse?
    - Safety planning may include working with families and authorities to find a safe placement for the child while investigations are taking place.

### Randomization and blinding

All participants were assigned a unique ID number following informed consent procedures. ID numbers of eligible participants were forwarded from the assessors to the Zambia-based study director at the end of each day, who then sent them to a designated US-based research staff member who was not involved in clinical activities. The US-based staff member maintained a computer-generated (via Microsoft Excel) 1:1 randomization sequence stratified by study compound and in blocks of 20 (i.e. each 20 assignments included 10 CETA and 10 TAU). Each of the three lists contained a sequence of treatment assignments in random order. These lists were not available or viewable to any staff in Zambia. The US-based research staff member would assign a family to the next available slot on the randomization sequence. Eligible families were contacted within a day of randomization to inform them of their status. Study assessors and data analysts were masked to randomization status at the baseline assessment and will remain blinded throughout the duration of the study.

### Interventions

#### CETA

CETA is a transdiagnostic mental health intervention developed for delivery by non-professionals in LMIC (Murray *et al.* 2014). The development of CETA (Table 1) was based on research of common elements or transdiagnostic treatment approaches used in the USA (Chorpita & Daleiden, 2009; Farchione *et al.* 2012; Weisz *et al.* 2012), but with a focus on being appropriate for training and delivery by non-professional (i.e. lay providers) in lower resource settings (Murray *et al.* 2014). CETA is not a ‘new’ treatment but rather an *approach* that teaches CBT elements common to evidence-based treatments (Chorpita *et al*. 2005) for trauma, anxiety, depression, and behavioral problems. As a CETA, this approach allows a counselor to flexibly decide on what element(s), order, and dose are needed depending on presentation.

**Table 1.** Components of CETA

Component	Simplified name (used in training)	Description
Psychoeducation and engagement	Introduction	Focus on obstacles to engagement Linking program to assisting with client's problems Includes family when appropriate Program information (duration, content, expectations) Normalization/validation of current symptoms/problems
Anxiety management strategies	Relaxation	Strategies to improve physiological stress Examples include: deep breathing, meditation, muscle relaxation, and imagery. Others added by local cultures
Behavioral activation	Getting active (GA)	Identifying and engaging in pleasurable, mood-boosting, or efficacy-increasing activities
Cognitive coping/restructuring	Thinking in a different way – part I and part II (TDW1 and TDW2)	Understand association between thoughts, feelings, and behavior Learn to restructure thinking to be more accurate and/or helpful
Imaginal gradual exposure	Talking about trauma memories (TDM)	Facing feared and avoided memories in detail Gradual desensitization/exposure
In vivo exposure	Live exposure	Facing innocuous triggers/reminders in the client's environment Gradual desensitization/exposure
Suicide/homicide/danger assessment and planning	Safety	Assessing client risk for suicide, homicide, and domestic violence Developing a focused plan with the client and client's family (when appropriate) Additional referral/reporting when needed
CBT for substance use and relapse prevention	Substance use element (SU)	Utilizes motivation and CBT principles and activities to get client buy-in and alter behavior patterns to change substance use/abuse behavior
Safety planning and violence prevention	Safety and violence prevention	Walks through detailed safety plans specific to IPV Discusses behavioral or situational modifications that may help prevent violence

CETA was chosen as the intervention due to its evidence base and its modular transdiagnostic design. CETA is currently the only transdiagnostic model with two rigorous clinical trials in LMIC that each show strong effect sizes across a range of symptoms: (a) on the Thailand/Myanmar border with Myanmar refugees (*N* = 347; ES: 1.19 post-traumatic stress, 1.16 depression, 0.79 anxiety) (Bolton *et al*. 2014) and (b) in Southern Iraq with survivors of conflict, torture, trauma, and ongoing stressors (*N* = 149; ES: 2.40 post-traumatic stress, 1.82 depression, 1.60 anxiety) (Weisz *et al*. 2015). These findings provide some evidence of effectiveness for CETA with adults, as well as ability of lay providers to learn this type of modular, flexible approach. In Ethiopia, results showed significant decreases in symptoms of internalizing (*d* = 1.37), externalizing (*d* = 0.85), and post-traumatic stress (*d* = 1.71) symptoms, and improvements in well-being (*d* = 0.75) (Murray *et al.* under review). The modular nature of CETA allowed us to develop and adapt the elements and flows to fit our study population.

**Fig. 2.**
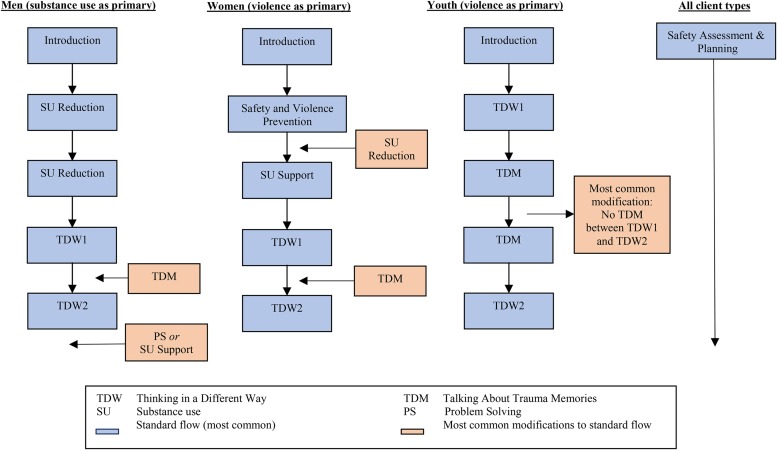
Most common flow of CETA intervention elements with possible modifications.

For this study, CETA was originally planned and initially implemented as a group treatment. Groups were set up to be specific to one participant type (i.e. men, women, or children) and composed of 5–7 individuals. Group assignment was based on the participants’ language, residence, and age (for youth groups). Each group was run by two trained providers pre-paired based on language fluency and location. Specifically, counselors were not allowed to work in or near the neighborhoods where they reside to reduce counselor bias and ensure client anonymity. Groups were designed to run for 90–120 min, with each session beginning with a tea time where participants could socialize to promote group cohesion and motivate regular and punctual attendance. Delivery of CETA included varying elements and ‘dose’ of elements (i.e. the number of sessions or time spent on it) depending on clients’ symptoms, with a suggested 6–12 weekly sessions depending on need. Figure 2 shows a common flow for the men's, women's, and children's group with comments on what might be added for flexibility based on need.

CETA was adapted for this trial to include a CBT-based substance use (SU) reduction element. CBT for SU disorders has demonstrated efficacy as both a mono-therapy and as part of combination treatment strategies (McHugh *et al.* 2010). Evidence from numerous large-scale trials and reviews support the efficacy of CBT for alcohol and drug use disorders (Dutra *et al.* 2008; Magill & Ray, 2009). Typically, CBT for SU includes any or a combination of the following: (a) motivational enhancement strategies, which target ambivalence to behavior change related to substances (Miller & Rollnick, 2013); (b) contingency management approaches, which work on countering the reinforcing effects of substances and providing positive reinforcements tied to non-using decisions; (c) relapse prevention, which applies a functional analysis of personalized SU cues (e.g. places, smells, people, etc. that promote urges to use) and implementation of alternative/competing responses to these cues.

Two authors drafted the SU element (LKM and CKD), which was then reviewed by other CETA trainers, SU treatment experts, and local counselors from Zambia who had provided other CBT treatments (i.e. trauma-focused CBT; Murray *et al.* 2015). Within the CETA SU element, there were motivational statements, including asking participants at multiple time points throughout the session to determine a behaviorally specific goal and rate their motivation to complete that goal. Adapted from other empirically supported SU treatments (Henggeler *et al.* 2002; Danielson *et al.* 2012), this component also includes helping participants identify all of the drivers that underlie their drinking behavior (e.g. boredom, passing the bar on their way home, coping with stress). Specific interventions and strategies that counter this list of individualized reasons listed (e.g. behavior replacement, avoidance) are then taught to the participants and practiced. The session is completed with revisiting the individual goals and the motivation to work toward or complete that goal. This component was designed in the context of this study to be delivered to all men, and also any women who also reported substance abuse.

Since our population of interest was the family unit, and our primary outcome is prevention of violence (which commonly co-occurs with SU; Widom & White, 1997) our team also developed a ‘SU support’ element that was delivered to the women when applicable. Utilization of a family support system, including family-based approaches, has been shown to be an efficacious approach to decreasing SU and relapse in other populations (Waldron & Turner, 2008). In CETA, the SU support element is designed to: (a) provide psychoeducation about the SU to the partner of the substance abuser, (b) to have the partner help in identifying the user's drivers (e.g. why the participant uses), and (c) to help the partner identify ways in which she can support the reduction of use (e.g. plan activities that are not at a bar, offer a favorite dinner with a beer at home).

The apprenticeship model of training and supervision is being used to deliver CETA (Murray *et al*. 2011). A 10-day in-person CETA training was conducted by study authors (LKM and SSVw), followed by weekly small group meetings in which lay counselors practiced the treatment elements with a local supervisor (before providing CETA to clients). Sixty-three lay counselors (20 male, 43 female) and seven supervisors (3 male, 4 female) between the ages of 20 and 60 years (average age 33.7 years) were trained in February and March 2016. Supervisors of CETA completed one pilot treatment group to strengthen their skills and understanding of the components decision-making process and CETA treatment. Counselors continue to meet in small groups and receive supervision on each case throughout the study. Weekly meetings are also continuously held between each local supervisor and a CETA trainer (SSVw in person, LKM via skype) for 2–3 h to either review group role-plays or discuss CETA cases.

#### Treatment as usual

The control condition is defined as TAU. In Zambia, no formal services or standard of care exists for IPV or alcohol use problems. There are some organizations that provide services such as informal counseling from parish priests, church officials, or other community leaders. At the beginning of the study, all participants were provided a list of relevant services in Lusaka. We did not exclude any families based on having received these types of services and do not in any way dissuade families from accessing these types of services or information during the study.

### Assessment of outcomes and follow-up

Post-assessments were conducted with participants starting in October 2016 and are ongoing. All post-assessments (4/5, 12, and 24 months post-baseline) are conducted in the same format (i.e. ACASI), and at the same locations as baseline. At baseline and all follow-up visits, participants are provided with reimbursement for travel. The primary outcome in the study is violence as measured by the SVAWS. Secondary outcomes include alcohol and other SU, mental health, psychological violence, and gender norms. Each participant type (woman, man, child) completes a specific battery of instruments via ACASI. The measures included in ACASI were pilot tested with participants sampled from the same source population as those in the trial to check translation accuracy and item comprehension. In addition to the self-report measures administered via ACASI, a hair sample is collected from participants as a biomarker of chronic stress (Russell *et al.* 2012). The full list of outcome instruments is displayed in Table 2.

**Table 2.** Primary and secondary outcome measures for VATU trial

				Cronbach's *α*^a^		
Measure	Construct	Reference	Description	Woman	Man	Child
Primary outcome						
Severity of Violence against Women Scale (SVAWS)	Violence	Marshall (1992)	46 items assessing how often (never, once, a few times, many times) a current partner perpetrated acts of violence against the participant in the past year. Subscales include: (a) threats of violence; (b) physical violence; and (c) sexual violence. Previously used in a study of IPV among women with partners who have alcohol abuse in South Africa (Peltzer & Pengpid, 2013)	0.95	–	–
Secondary outcomes						
World Health Organization IPV measure (WHO-IPV)^b^	Violence	World Health Organization (2005)	Nine items assessing how many times a current or previous partner ever and recently perpetrated acts of violence (never, once, a few, many). Previously used in the WHO multi-country study on Women's Health, including in Tanzania and Namibia	0.95	0.96	–
Youth Victimization Scale (YVS)	Violence	Nadel *et al*. (1996)	135 items assessing how often (never, once, sometimes, often) youth have experienced various types of recent violence at school, in the neighborhood, and at home	–	–	0.98
Alcohol Use Disorders Identification Test (AUDIT)^c^	Alcohol	Saunders *et al.* (1993)	10 items assessing hazardous alcohol use. Scores of ⩾3 for women and ⩾4 for men are considered hazardous. Previously validated in Zambia (Chishinga *et al.* 2011)	0.88	0.85	–
Alcohol, Smoking, and Substance Involvement Screening Test (ASSIST)	Drug use	Humeniuk *et al.* (2008)	Seven items assessing frequency and consequences of use for a range of substance types. Previously validated in Zambia (Kane *et al.* 2016*a*)	0.97	0.97	0.98
Center for Epidemiological Studies-Depression Scale (CES-D)	Depression	Radloff (1977)	20 items assessing frequency of depression symptoms over the past week (never, 1–2, 3–4, 5–7 days). Previously validated in Zambia (Chishinga *et al.* 2011)	0.92	0.90	–
Youth Self Report	Mental/behavioral health	Achenbach (1991)	112 items assessing occurrence of child mental health symptoms and behaviors in the past 4 weeks. Response options are: not true, sometimes true, very true. Subscales assess internalizing symptoms and externalizing behaviors. Validation study among adolescents in Zambia currently underway as part of an ongoing study (clinicaltrials.gov # NCT02054780)	–	–	0.98
Harvard Trauma Questionnaire (HTQ)	Trauma/post-traumatic stress disorder (PTSD)	Mollica *et al.* (1992)	17 items assessing lifetime experience of a range of traumatic events. 39 items assessing symptoms of post-traumatic stress in the past week (not at all, a little, quite a bit, extremely)	0.96	0.95	–
Child PTSD Symptom Scale (CPSS)	Trauma/PTSD	Foa *et al*. (2001)	14 items assessing lifetime experience of a range of traumatic events. 17 items assessing symptoms of post-traumatic stress in youth in the past 2 weeks (never, once in a while, more than half the time, almost always). Validation study among adolescents in Zambia currently underway as part of an ongoing study (clinicaltrials.gov # NCT02054780)	–	–	0.93
Aggression Scale	Aggression	Orpinas & Frankowski (2001)	11 items assessing frequency (range of 0 times to 6+ times) of youth aggressive behaviors over the past week	–	–	0.87
Gender Equitable Men's Scale (GEMS)	Gender norms	Pulerwitz & Barker (2007)	24 items assessing the degree to which adult participants agree (agree, partially agree, do not agree) with statements on gender norms	0.88	0.89	–
Index of Psychological Abuse (IPA)	Psychological violence	Sullivan & Bybee (1999)	33 items assessing how often a partner perpetrated psychological abuse over the past 3 months (never, rarely, sometimes, often)	0.94	–	–
Hair sample for cortisol biomarker	Chronic stress	Henley *et al.* (2013)	–	–	–	–

a Cronbach's *α* calculated from all available baseline data

b The woman reports *experiencing* violence and the man reports *perpetration* of violence on the WHO-IPV measure

c Participants complete two versions of the AUDIT; one in reference to their own alcohol use and one in reference to their partner's alcohol use

In addition to the primary assessment time points, participants are tracked weekly during the intervention phase. For CETA participants, counselors administer a brief symptom monitoring form and the Alcohol Timeline Followback (TLFB; Sobell & Sobell, 1992) at each session. Additionally, the Short Inventory of Problems (SIP; Tonigan & Miller, 2002), which measures consequences associated with alcohol misuse, is administered at the first and last CETA session. Control participants receive weekly phone calls or home visits from our research staff to assess safety during the intervention period and complete the Alcohol TLFB and brief symptom monitoring form monthly. Following the first ACASI post-assessment, both control and intervention participants receive monthly safety check-in phone calls from the research team.

### Data and safety monitoring

The trial is monitored by a four-person Data Safety Monitoring Board (DSMB). It was mutually determined between DSMB members and study investigators that if the CETA intervention displayed statistically significant effectiveness with at least a moderate effect size at the 12-month follow-up assessment, it would be ethically appropriate to stop the trial and offer the intervention to control participants. Therefore, the DSMB will conduct an effectiveness analysis following the completion of the 12-month post-baseline assessments. The trial will be unmasked and stopped if there is a statistically significant difference between the change in primary outcome (i.e. SVAWS score among female participants) by treatment arm, defined as a Cohen's *d* effect size of ⩾0.5 and a *p* value of <0.05. In that scenario, control participants would not be assessed at 24 months post-baseline but would be offered CETA, and CETA participants would still be assessed at 24 months as planned to measure whether treatment effects were sustained. If no significant difference in change in SVAWS score by treatment arm is found in the 12-month DSMB analysis, the trial will continue as planned with all participants assessed at the primary study endpoint of 24 months (see Fig. 1).

### Data analysis

Sample size calculations were informed by a study conducted in South Africa that used the SVAWS physical violence subscale to measure violence among women who had been abused by a partner with an alcohol use problem (Peltzer & Pengpid, 2013). We calculated the sample size needed to obtain a 20% reduction in SVAWS physical violence subscale score among intervention participants at a 24-month follow-up, assuming no change among control participants, using a two-sample independent means *t* test. For 80% power at an *α* level of 0.05, we calculated that we would need to enroll a minimum of 50 families in each study arm to detect this difference in change in means, which we inflated to 84 per arm to account for loss to follow-up and the possibility of small clustering effects at the provider level.

All primary analyses will be based on an intent-to-treat approach with all participants who were randomized included in the final analysis. Loss to follow-up and missing data will be addressed by using multiple imputation (Azur *et al.* 2011). We will estimate linear (continuous outcomes) and generalized linear (binary outcomes) mixed-effects models that will incorporate a random intercept term to account for within-subject correlation on repeated measures. The outcome scores at the three follow-up times will be modeled separately using treatment arm, baseline symptom score (if there is a meaningful difference between the study arms at baseline), time, and an interaction between each treatment arm and time as fixed covariates in each model.

### Modification to study protocol after initiation of the trial

We modified the delivery of CETA from group to individual. After the first few weeks of intervention delivery, multiple participants were missing group sessions due to logistical challenges (e.g. work, funerals), which necessitated CETA providers to conduct separate individual sessions for participants that were absent. It became challenging for providers to keep up with the many in-between group sessions they had to conduct, and then also had to repeat material in groups if an individual missed and was not available in-between group sessions. Participants also indicated frustration in that they did want to participate but there was no flexibility for tardiness (in Zambia, this may be defined as an hour or more late) or work/family scheduling within groups. The challenges were substantial enough that we would not recommend group CETA in Lusaka, Zambia (urban area), even if it was found to be clinically effective. We therefore modified CETA to be individually delivered. Participants initiating CETA before this change could switch to receiving individually delivered CETA or continue to attend group sessions based on their preference and the counselor's assessment of feasibility.

We increased our sample size because of this modification to the delivery of CETA. We plan to conduct a subgroup analysis of only those study participants who received individual (and not group) CETA to test whether this individual delivery mode is effective compared with the TAU control group. Of the 83 families originally randomized to CETA, 33 men and 40 women did not receive a group session (only individual session or sessions). Our original sample size calculation for the CETA arm was *n* = 50. To have at least *n* = 50 CETA participants who received individually delivered therapy, and taking into account potential for up to 30% loss to follow-up, we recruited and randomized an additional 80 families (again randomizing using 1:1 allocation). The final sample size for the study is thus *n* = 248. All the families recruited in this second cohort that were randomized to the CETA arm are receiving individually delivered CETA.

### Trial status

Recruitment, screening, and randomization of all study participants were completed in December 2016. The first wave of recruitment occurred between May and July 2016 and the second wave (following the sample size increase) occurred between November and December 2016. Participant baseline characteristics are summarized in Table 3. All participants are expected to complete the CETA intervention and first post-assessment by April 2017. The 12-month post-assessment will occur between May and July 2017 for the first cohort of families recruited and between November and December 2017 for the second cohort. The 24-month post-assessment will occur between May and July 2018 and between November and December 2018 for the first and second cohorts, respectively.

**Table 3.** Baseline participant characteristics

	Adult female (*n* = 248)	Adult male (*n* = 248)	Child (*n* = 130)
Age			
18–25	65 (26.2)	27 (10.9)	
26–35	99 (39.9)	94 (37.9)	8–9 28 (21.5)
36–45	49 (20)	74 (29.8)	10–11 33 (25.4)
46–55	25 (10.1)	37 (14.9)	12–13 30 (23.1)
56–65	7 (2.8)	11 (4.4)	14–15 20 (15.4)
66+	2 (0.8)	5 (2.0)	16–17 19 (14.6)
Female	–	–	70 (53.9)
HIV positive	101 (40.7)	66 (26.6)	12 (9.2)
SVAWS physical violence subscale, mean (s.d.)	60.7 (16.8)	–	–
Child abuse (physical)^a^	–	–	54 (41.5)
Child abuse (verbal/emotional)^b^	–	–	49 (37.7)
AUDIT (self-report), mean (s.d.)	10.7 (11.0)	15.8 (10.4)	–
Lifetime substance use^c^	116 (47.0)	159 (64.4)	–
Harvard Trauma Questionnaire (HTQ), mean (s.d.)	1.5 (0.7)	1.3 (0.7)	–
Child PTSD Symptom Scale (CPSS), mean (s.d.)	–	–	0.7 (0.7)
Center for Epidemiological Studies-Depression (CES-D), mean (s.d.)	2.2 (0.7)	2.0 (0.6)	–

Values are *n (%)* unless otherwise specified and based on all available data at baseline

a Response of once, sometimes, or often, to item ‘how often have you been beaten at home in the past 12 months’

b Response of once, sometimes, or often to item ‘how often have you been verbally or emotionally abused at home in the past 12 months’

c Any lifetime substance use reported not including alcohol or tobacco on the Alcohol, Smoking, and Substance Involvement Screening Test

## Discussion

To our knowledge, this is the first trial to test the effectiveness of a modular transdiagnostic treatment approach on VAWG among families in LMIC by addressing risk factors for experiencing violence (among women and children) and perpetrating violence (among men). Given that many of the violence prevention strategies assessed in LMIC are community-based structural approaches (Bourey *et al.* 2015), the findings of this trial will enhance understanding of the utility of a targeted treatment approach among high-risk populations (i.e. those with recent severe IPV and hazardous alcohol use), and thus inform policy decisions. Effective combinations of structural (e.g. microfinance, legislation, social empowerment community programs) and clinical (e.g. CBT, CETA) strategies warrant future research. The study has already yielded important information about the clinical challenges associated with group-based therapy in urban LMIC settings.

The study design is not without limitation. Child outcomes from this trial will be considered exploratory. For ethical reasons, we did not exclude families without children, those with children who were infants or grown adults, or those that did not want a child to participate in the study. This resulted in a relatively small number of youth (*n* = 130; 52.4% of recruited families) being enrolled and the study not being powered to assess change in child outcomes. If the results of these exploratory analyses are promising, future trials should include a larger sample of children, when possible, to better ascertain the effectiveness on family members.

Despite this limitation, our study design is characterized by several important strengths. This trial uses the most robust design feasible in Zambia to measure the effectiveness of CETA: randomization with masking at the assessor level, ACASI-administered assessments, a TAU comparator, and multiple outcome time points with an extended follow-up period relative to many previous investigations of violence interventions in LMIC (Bourey *et al.* 2015). The primary violence outcome is based on an indicator of violence severity (SVAWS), which enhances the statistical power of the study and provides a more nuanced measure of violence experiences than more commonly used binary indicators. Finally, we have instituted an intensive safety plan for study participants, which may be useful for future research with similarly high-risk populations.

We believe that this study has the potential to be generalizable to other low resource settings with populations affected by violence and/or SU. Similar to most other LMIC, Zambia does not have a mental health infrastructure where providers are trained in evidence-based methods to work with violence and alcohol abuse. It is likely that most LMIC would use lay providers similar to this study if they were to replicate the intervention, and that there would be limited other services available to address these problems. Further, violence and alcohol abuse cut across all cultures and socioeconomic levels – and are very commonly comorbid. In fact, estimates of alcohol use and violence have been reported at high levels across the sub-Saharan Africa region (UNODC, 2012; World Health Organization, 2013). Finally, the trial has few exclusion criteria, which will increase the generalizability of findings.

In closing, we believe that the VATU trial will fill a critical gap in current knowledge on the effectiveness of interventions for VAWG in resource-limited settings. Given the significant morbidity and mortality associated with VAWG, rigorously designed effectiveness studies of prevention and reduction strategies are urgently needed. The VATU trial is part of the *What Works* consortium of studies that currently includes eight impact evaluations across Africa, Asia, and the Middle East. In addition to executing the research, a significant effort of the program is to build capacity of local partners in research design and intervention delivery. Taken together, the capacity building efforts and findings from these ongoing investigations have the potential to inform programming, policy, and research on impactful and cost-effective intervention approaches to reducing VAWG among families in LMIC.

## Supplementary material

For supplementary material accompanying this paper visit http://dx.doi.org/10.1017/gmh.2017.10. click here to view supplementary material
