# Supplementary material for: Testing the effectiveness of a transdiagnostic treatment approach in reducing violence and alcohol abuse among families in Zambia: study protocol of the Violence and Alcohol Treatment (VATU) trial
Source: Glob Ment Health (Camb). 2017 Oct 2;4:e18. doi: 10.1017/gmh.2017.10 (PMC5719477; doi:10.1017/gmh.2017.10)
Supplement: Supplementary file 1 [file S2054425117000103sup001.docx]

**Safety Plan**

**PURPOSE**

The purpose of this plan is to ensure the safety and well-being of families in our study to safeguard them against any potential abuse or self-harm. They will also safeguard the staff and participants from allegations that might arise from careless or unwise behavior. It also describes what steps are to be taken if an adult or child in the study is suicidal, homicidal, threatens violence, is a victim of domestic violence, and/or is being sexually or physically abused.

**SCOPE**

This procedure applies to all staff involved in the Randomized Controlled Trial of a Multi-pronged Intervention to Address Prevention of Violence in Zambia (VATU)

**RESPONSIBILITIES**

All project staff whether full time or part time who talk with participants in this study are responsible for understanding and following this plan. The project manager has ultimate responsibility for ensuring that all applicable staff follows this plan.

**DEFINITIONS**

Safe homes - These are organizations that will provide safe housing to women and children who need a safe place to stay.

Physical abuse – One or more episodes of aggressive behavior, usually resulting in physical injury with possible damage to the internal organs, sensory organ, the central nervous system, or the musculoskeletal system of another person.

Homicide- The killing of a person by another, regardless of intent.

Threatening Violence – When a participant says they will intentionally and physically harm another person, either directly to the person threatened or to another person.

Suicide – The intentional taking of one’s own life.

Domestic violence – When children witness physical aggression between adults living in the home.

Sexual abuse – Also referred to as defilement, this is the forcing of the undesired sexual behavior by an adult onto a child or participant. Whether that is immediate, of short duration or infrequent.

**PROCEDURES**

**Assessing risk for SUICIDE**

All study staff must follow these steps when there is concern that the participant in the study has intentions of killing him or herself. When assessing for the risk of suicide the staff should ask the participant the following questions

1. “Do you think about killing yourself?”
2. “Do you have a plan to kill yourself?”
3. “Do you have a way to carry out this plan?”
4. “Have you ever tried killing yourself?”

When the staff asks question 3,

- S/he tries to find out if the participant has access to medicine, a gun, gas stove, or other means of killing him or herself. It is very important to know if a participant who is thinking about suicide has immediate access to a way to commit suicide.

When the staff asks question 4,

- S/he tries to find out if the participant has ever tried killing themselves before; they are at even higher risk.

The staff will ensure to purposely ask these questions in a direct way because suicide is a very serious, sometimes life/death situation. The staff will then develop a safety plan if the participant responds with a “yes” to any of these questions.

**If the participant responds “yes” to ANY QUESTIONS the staff should call one of the clinical supervisors while the participant is still with them**. The staff should remind the participant of the limits of confidentiality and tell them that you need to call your supervisor to help keep them safe.

- The staff should talk with the supervisor about what the participant reported and the specific safety concerns about this participant AND go over the safety plan that was developed with the participant.
- The staff will check with the supervisor if there is anything else that needs to be added to the safety plan or done to keep the participant safe
- The staff will also check if there is anything else that needs to be followed up on with the participant quickly (before the next session or contact)
- If it is recommended the staff may need to escort the client to the psychiatric hospital for further evaluation.
- If the participant refuses to go to the clinic, ask the participant to wait as the staff contacts Supervisor again from another room and explain the case of threatened suicide. Ask the supervisor to come and accompany the client to the clinic.

If the supervisor cannot be reached the staff should contact the clinical study director and follow the steps below:

Developing a safety plan for suicide

- The staff will talk to the participant about **what** s/he intends to do with the participant (that is develop a safety plan for them).
- The staff will normalize the participants feeling by saying “many people have thoughts of killing themselves”
- The staff will then tell the participant that there are many things that can be done to help the participant feel better and also help the participant stay safe.
- The staff will then tell the participant **why** it is important that they keep him or her safe. The staff will explain to the participant that the treatment the participant is undergoing has helped many people with their feelings of sadness, anxiety, and wish to die.

Possible step by step actions that the staffs can take

1. Telling a trusted family member

- The staff will try to involve a family member or friend that the participant trusts (such as parents, adult sibling, or another adult family member). This will ensure that another person knows the safety concerns of the participant and also how to help the participant stay safe.
- The staff will ensure the family member or friend being contacted or being called in the session is one the participant trusts (and not a person who is hurting the participant).
- The staff should ensure that s/he meets with the participant and the family member or friend that day or the next day as part of follow up. This is to develop a plan and safety support for the participant.

1. With the participant identify warning signs

- The staff should ask the participant the following questions:
- What are some of the signs you have that trigger thoughts of killing yourself?
- How do you feel?
- What do you do?
- What are you thinking?
- What are the situations when you are most likely to think about killing yourself?

1. Help the participant use their skills

- The staff will encourage the participant to use their skills, which could be things they already do and/or new things they have learned from the program.

- The staff will then ask the participant the following questions:
  - When you start thinking or feeling or doing these things that trigger thoughts of suicide, what are some of the things you do or think to help decrease the thoughts about wanting to die?
  - Are there people you talk to or activities that you do to lessen the thoughts or distract yourself from the thoughts?
  - Among these people you have mentioned or activities you have mentioned, which of these can you do more of or keep doing?
- The staff should talk to the participant about the skills s/he has learned in this or other programs they may have participated in. For example staff can ask the participant *“Are there things you have done in the past when you have felt this way that have helped you feel better?”*
- The staff shall make a list or review the skills the participant has and can use to prevent or reduce the impact of suicidal thoughts. The staff will thereafter ask the participant the following questions:
  - *“How will you know when the safety plan that we will develop should be used?”*
  - *“What can you do, on your own, if you [become suicidal again/ have urge to drink/hurt yourself], to help yourself not to act on thoughts or urges?”*
  - *“How likely is it that you would be able to do some of these things during a time of crisis?”*
- If the participant expresses doubt about use, ask “*What might get in the way of you thinking of these activities or doing them if you think of them?”*

1. Develop a contract for safety

- The staff will develop a note together with the participant that states that the participant promises or gives their word that they will keep themselves safe and will not kill themselves for a short period of time (e.g., 24 hours). If they do feel like or think about killing themselves, the note will contain specific instructions of who to call or where they can go to stay safe.
- “We want to make sure you are safe. I know this might be hard. Can you give me your word that you will keep yourself safe for a short period of time – just over the next day?”

1. Develop a safety watch

- If the participant has someone they are around often who is willing to help, the staff can ask this person to “watch” the participant. It is arranged that the participant is never by him/herself, but is always surrounded by at least one family member or friend.
- This is set up for short periods of time (a few days).
- “We want to help you keep yourself safe. Many times we use family members to do this. Can you help me think of who in your family can be around you?”
- “Can we work together to bring these family members in to agree to help be with you so that you stay safe?”

Plan for follow up

- A plan for more frequent visits and/or follow ups should be put into place if a participant expresses suicidal thoughts with a plan or there is real concern that the participant will be unable to keep the contract for safety. This can take the form of brief check-ins over the phone or at the office. If a family member or friend whom the participant trusts is identified, this adult can be included in these follow up visits and contacts.
- Follow-ups should take place more frequently right after the disclosure of suicidal thoughts (once a day for 3 days) and then slowly decrease as the concern for safety decreases.

**Assessing risk of HOMICIDE/THREATENING VIOLENCE**

The staff should ask the following questions:

1. Have you ever tried to end someone’s life or hurt someone before?
2. Are you thinking of ending someone’s life or hurting someone?
3. Do you have a plan to end someone’s life or hurting that person?
4. Do you have access to that person? Do you have the means to execute your plan?

If a client answers “YES” to any of the following questions, immediately inform the clinical supervisors. The staff will follow the steps below:

1. The staff and the participant will agree together that if the participant has thoughts of killing anyone, the participant will speak to the staff personally before carrying out any plans to harm anyone.
   1. The staff will ask the participant if s/he will be able to speak with the staff if/when s/he has such thoughts.
2. If the participant says "no" or "I don't know," to the questions 3 and 4, the staff will ask the participant the following questions:
   1. The staff will rephrase what the participant is saying for example
   2. "What I am hearing is that you are in a lot of pain right now and thinking of ending someone’s life, so I want you to come with me right now to get some help to make you feel better. I will make sure you get there safely. Is there a family member or someone I can call to go with you?"
3. The staff will arrange for the person to be accompanied to a safe place, and will call her/his supervisor ahead to advise the supervisor of the situation.
4. If the participant refuses, the staff will ask the participant to wait at the location while the staff calls the supervisor in another room to report that the person has threatened homicide/violence. The staff will ask the supervisor what to do next.

**Assessing risk of DOMESTIC VIOLENCE:**

To assess risk, the staff will follow the study safety protocol and clearly ask the following three questions:

1. Is the perpetrator of domestic violence living with you?
2. In what ways has this person hurt you?
3. In what ways do you think this person could hurt you?

The staff will talk to her/his supervisor while the participant is still working with them. The staff and the participant will decide or agree on a plan BEFORE the participant leaves the session.

The staff will work with the participant and supervisor to develop an action plan. The action plan will follow the guidelines of the Zambian Anti-Gender Based Violence Act of 2011 which states:

“A police officer, labour inspector, social worker, counsellor,

medical practitioner, legal practitioner, nurse, religious leader,

traditional leader, teacher, employer or other person or institution

with information concerning the commission of an act of gender based

violence shall—

(a) inform a victim of the victim’s rights and any basic support

which may be available to assist the victim;

(b) obtain for the victim, or advise the victim how to obtain

shelter, medical treatment, legal services, counseling or

other service that may be required in the circumstances;

and

(c) advise the victim of the victim’s right to lodge a complaint

against the respondent including remedies available to

the victim under this Act.”

All staff will provide adult victims of domestic violence with 1) a copy of the full Zambian Anti-Gender Based Violence Act 2) a resource list that includes local shelters, legal, medical and counseling services and contact information for other services that may be needed 3) assistance in reporting or lodging complaints against the perpetrator at the victims request.

If the participant is a child and answers “yes” to witnessing severe violence in the home as measured by the Child Victimization Scale, immediately the staff will call one of the clinical supervisors. The clinical supervisor may then advise the staff to either contact child protection officers or to contact one of the social workers at the social welfare office if there is any uncertainty that the case should be reported to the police. In addition to discussing the participant’s risk situation with the clinical supervisor and police, the staff should also ensure that they have formulated a safety plan with the participant.

Find below a checklist to help review what the staff must attend to. The action plan could include the following checklist:

- Has the staff reported the incident to the child protection officer?
- Has staff notified the supervisor?
- Has staff developed a safety plan with the participant?

The child protection officer must report to the Child Protection Unit at the Ministry of Gender and Child Development. If necessary, the child protection officers will assist in getting the child to a temporary safe placement home until investigations are concluded.

**If you do not succeed in getting in contact with your supervisor, in this specific case, immediately contact the clinical study director.**

**Assessing risk for CHILD SEXUAL OR PHYSICAL ABUSE:**

To assess risk, the staff should clearly ask the following four questions:

1. Is the abuse currently happening (i.e. within the past weeks or month)?
2. Do you know the person who is abusing you?
3. Does the abuser stay in the same house with you?
4. Have you reported the abuse to anybody?
5. If you have reported the abuse, what has been done?

If the client answers “YES” to the first three questions, immediately call designated contacts at the Ministry of Gender and Child Development. The staff shall ensure to call their supervisor at the end of the session.

If the client says no to the first questions ask for more information. For example:

- When was the last time the abuse happened?
- Where is the person who was abusing you currently staying?
- Does anyone in the home know about the abuse?

If you are not certain if this case should be reported to the Child Protection Unit, please contact your clinical supervisors who will instruct you on who to contact (you may also need to contact the Social Welfare Office in cases where there is a question about reporting).

Below are certain things to do during the session.

In addition to formulating a safety plan, use the following checklist to review what has been done:

- Have you informed your supervisor?
- Did you inform the child protection officer?
- Has the child protection officer reported to the Child Protection Unit who will get the child to safe temporary placement home until investigations are concluded? If child’s life is proven to be in danger, we will work with CPU and the police in hopes of arresting and prosecuting the perpetrator. We will also follow up with CPU and the family to ensure the child remains in a safe environment.

**If you do not succeed in getting in contact with your supervisor immediately contact the clinical study director.**
